# The unicuspid aortic valve

**Published:** 2010-04

**Authors:** SHI-MIN YUAN, JACOB LAVEE, SHI-MIN YUAN, HUA JING

**Affiliations:** Department of Cardiac and Thoracic Surgery, The Chaim Sheba Medical Center, Tel Hashomer, Israel; Department of Cardiac and Thoracic Surgery, The Chaim Sheba Medical Center, Tel Hashomer, Israel; Department of Cardiothoracic Surgery, Jinling Hospital, School of Clinical Medicine, Nanjing University, Nanjing, Jiangsu Province, People’s Republic of China; Department of Cardiothoracic Surgery, Jinling Hospital, School of Clinical Medicine, Nanjing University, Nanjing, Jiangsu Province, People’s Republic of China

**Keywords:** aortic dilatation, echocardiography, unicuspid aortic valve

## Abstract

The unicuspid aortic valve is a very rare congenital anomaly, which usually presents as aortic stenosis, incompetence, or a combination of both. Other congenital disorders may accompany this phenomenon and aortic dilatation and left ventricular hypertrophy are frequent complications. We present a case report of a young, symptomatic patient with a unicuspid aortic valve, complicated by dilatation of the aortic root and ascending aorta, with left ventricular hypertrophy. The patient recovered fully after a Bentall procedure.

## Introduction

The unicuspid aortic valve is a rare congenital cardiovascular anomaly, which is often misdiagnosed as a bicuspid aortic valve.^1^ The true incidence of the unicuspid aortic valve may be underestimated in the asymptomatic population.^2^ The clinical and diagnostic implications of this anomaly have been reviewed before.^2^-^6^

## Case Report

**Fig. 1.**
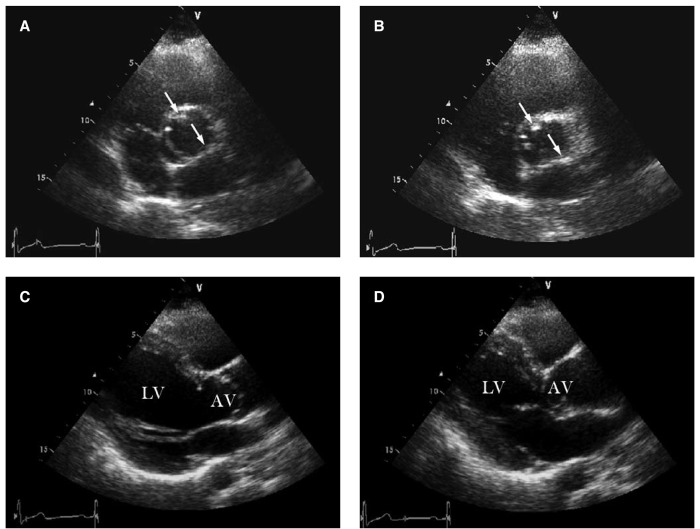
Transthoracic echocardiaography showing a unicuspid aortic valve with a raphe at the 11 o’clock position (upper arrow) and a clear commissure at the 4–5 o’clock position (lower arrow) on a short-axis view during systole (A), and diastole (B). The aortic valve in an integral movement and in a dome-shaped configuration during systole (C) and diastole (D), and left ventricular hypertrophy and dilated aortic root extending 3.8 cm in diameter could be seen from the parasternal long axis view (C, D). AV : aortic valve; LV : left ventricle.

These images (Fig. 1A-1D) are those of a 32-year-old male who presented with intermittent chest pain. Clinically, a combined systolic and diastolic murmur was audible over the left parasternal region. Chest radiography demonstrated a dilated ascending aorta. Echocardiography additionally revealed a unicuspid aortic valve – with one raphe and commissure. Severe aortic regurgitation with mild aortic stenosis, resulting in left ventricular hypertrophy was also present.

At operation, the aortic valve was unicuspid and severely regurgitant with an eccentric orifice, with one commissural attachment at the left- and non-coronary commissural, and one raphe at the right- and left-coronary commissural positions, with leaflet thickening and calcification. Aortic dilatation involving the aortic root and ascending aorta was an additional operative finding. The patient underwent a Bentall procedure and had an uncomplicated post-operative course.

## Discussion

The unicuspid aortic valve is a rare congenital malformation seen in 0.019% of patients during echocardiographic evaluation and in 5.59% of patients during aortic valve replacement.^2^,^3^ The unicuspid aortic valve can be categorised into two types: acommissural pin-hole shaped, and unicommissural slit-shaped.^1^ The acommissural type has no lateral attachment to the aorta with a central orifice, and the unicommissural type has one attachment with an eccentric orifice.^3^

Patients with a unicuspid aortic valve are always very young at the time of diagnosis or surgery, ranging from 14 to 75 years old.^1^,^4^ The typical age of unicuspid aortic valve patients at presentation is the third to the sixth decade, indicating an earlier onset and a higher rate of progression of aortic stenosis in comparison to patients with a tricuspid aortic valve. Collins et al.^5^ have shown in a retrospective analysis that a decreased number of aortic cusps are associated with an increased occurrence of pathological changes of these cusps and the ascending aorta.

Severe aortic stenosis or mixed stenosis and regurgitation is the predominant disorder that accompanies patients with a unicuspid aortic valve.^6^ Left ventricular dilatation might be present at the time of diagnosis.^7^ Similar to the bicuspid aortic valve, the unicuspid aortic valve is prone to be associated with dilatation or dissection of the aorta, involving the aortic root,8 ascending aorta,^9^ or aortic arch,^10^ which typically requires surgical intervention. Other associated disorders include aortic coarctation, an aberrant right subclavian artery,^1^ and a single coronary artery and ventricular septal defects.^8^

Recently, magnetic resonance imaging, cardiac computed tomography, and multislice tomography angiography were also applied as auxiliary diagnostic tools in such patients by virtue of their promising assessment of aortic valve morphology, including the exact morphology of the aortic valve and the severity of the aortic stenosis and regurgitation.^1^,^11^,^12^ However, echocardiography remains a reliable method for the pre-operative diagnosis of a unicuspid aortic valve, preferable to the radiological diagnostic tools mentioned above.

Echocardiographic imaging allows diagnostic accuracy of aortic valve morphology in most patients. The commissural attachment zone, the valvular orifice, the free edge of the leaflet, and the configuration of the aortic valve can be clearly visualised. Besides, echocardiography can even distinguish true from false unicuspid aortic valves.^2^

Aortic valve repair, including bicuspidisation, can be performed with low risk and excellent operative results.^9^

## References

[1] Dursun M, Yilmaz S, Sayin OA, Ugurlucan M, Ucar A, Yekeler E, Tunaci A (2007). Combination of unicuspid aortic valve, aortic coarctation, and aberrant right subclavian artery in a child: MR imaging and CTA findings.. Cardiovasc Intervent Radiol.

[2] Novaro GM, Mishra M, Griffin BP (2003). Incidence and echocardiographic features of congenital unicuspid aortic valve in an adult population.. J Heart Valve Dis.

[3] Falcone MW, Roberts WC, Morrow AG, Perloff JK (1971). Congenital aortic stenosis resulting from a unicommisssural valve. Clinical and anatomic features in twenty-one adult patients.. Circulation.

[4] Roberts WC, Ko JM (2007). Clinical and morphologic features of the congenitally unicuspid acommissural stenotic and regurgitant aortic valve.. Cardiology.

[5] Collins MJ, Butany J, Borger MA, Strauss BH, David TE (2008). Implications of a congenitally abnormal valve: a study of 1025 consecutively excised aortic valves.. J Clin Pathol.

[6] Singh D, Chee TS (2008). Incidental diagnosis of unicuspid aortic valve in an asymptomatic adult.. J Am Soc Echocardiogr.

[7] Murphy BA, Groban L, Kon ND (2003). Diagnosis of a unicuspid aortic valve using transesophageal echocardiography.. J Cardiothorac Vasc Anesth.

[8] Ishigami H, Iwase M, Hyoudo K, Aoyama I, Ito M, Tajima K (2005). A case of unicuspid aortic valve associated with a single coronary artery and ventricular septal defect.. J Med Ultrason.

[09] Schäfers HJ, Aicher D, Riodionycheva S, Lindinger A, Rädle-Hurst T, Langer F, Abdul-Khaliq H (2008). Bicuspidization of the unicuspid aortic valve: a new reconstructive approach.. Ann Thorac.

[10] Bansal A, Arora S, Traub D, Haybron D (2008). Unicuspid aortic valve and aortic arch aneurysm in a patient with Turner syndrome.. Asian Cardiovasc Thorac Ann.

[11] Debl K, Djavidani B, Buchner S, Poschenrieder F, Heinicke N, Schmid C (2008). Unicuspid aortic valve disease: a magnetic resonance imaging study.. Rofo.

[12] Gibbs WN, Hamman BL, Roberts WC, Schussler JM (2008). Diagnosis of congenital unicuspid aortic valve by 64-slice cardiac computed tomography.. Proc (bayl Univ Med Cent).

